# Comparing the capacity of five different dietary treatments to optimise growth and nutritional composition in two scleractinian corals

**DOI:** 10.1371/journal.pone.0207956

**Published:** 2018-11-28

**Authors:** Jessica A. Conlan, Line K. Bay, Andrea Severati, Craig Humphrey, David S. Francis

**Affiliations:** 1 School of Life and Environmental Sciences, Deakin University, Waurn Ponds, Victoria, Australia; 2 Australian Institute of Marine Science, Townsville, Queensland, Australia; Biodiversity Research Center, TAIWAN

## Abstract

Developing an optimal heterotrophic feeding regime has the potential to improve captive coral growth and health. This study evaluated the efficacy of three exogenous diets: *Artemia* nauplii (ART), a commercially available coral diet (Reef Roids) (RR), and a novel, micro-bound diet (ATF), against a comparatively natural, unfiltered seawater treatment (RAW), and an unfed, ultra-filtered seawater treatment (CTL), in adult *Acropora millepora* and *Pocillopora acuta* nubbins. After 90 days, both species showed significantly positive weight gain in response to one treatment (*A*. *millepora*–RAW, *P*. *acuta*–ART), and comparatively low growth in response to another (*A*. *millepora*–ATF, *P*. *acuta*–RR). The results highlighted substantial differences in the nutritional requirements between species. The nutritional composition of *A*. *millepora* in the best performing treatment was dominated by high-energy materials such as storage lipids and saturated and monounsaturated fatty acids. In contrast, the *P*. *acuta* nutritional profile in the superior treatment showed a predominance of structural materials, including protein, phospholipids, and polyunsaturated fatty acids. This study demonstrates that *Artemia* nauplii can successfully replace a natural feeding regime for captive *P*. *acuta*, yet highlights the considerable work still required to optimise supplementary feeding regimes for *A*. *millepora*.

## Introduction

The international aquarium trade is a multimillion dollar industry (US $200–330 million year^−1^) [1] in which live corals constitute highly popular ornamental marine species [2]. Consequently, significant efforts have been made in recent years to develop robust coral aquaculture practices that will enable mass coral production to support the growing aquarium trade [3]. Importantly, this will also provide sustainable coral stock for reef rehabilitation efforts and scientific research [4]. However, although coral breeding and propagation in captivity is a well-known activity among public aquariums and aquarium hobbyists, there remains a need for rigorous scientific research in this field to optimise current practices and maximise results [3].

*Ex situ* coral cultivation enables the manipulation of a closed environment to achieve optimal physico-chemical conditions, namely temperature, water quality, and lighting, as well as biological aspects such as food availability, whilst also avoiding natural stressors such as predation, sedimentation, and pathogens [3,5]. Attempts to culture coral in captivity have been carried out over several decades [3]. Today, the majority of zooxanthellate corals can be asexually propagated, and numerous species are successfully maintained in captivity [6].

Sexual reproduction is also becoming increasingly common in aquariums, although achieving synchronised mass-spawning of broadcast species is still highly uncommon and largely unpredictable [6].

Several aspects of coral aquaculture must still be optimised for the industry to prosper. These include optimising water chemistry, water flow, and lighting [3,7], as well as decreasing the transfer of pathogens and parasites [6]. In particular, satisfying coral nutritional requirements remains a critical task in mass-producing healthy coral and ultimately closing their life cycles to establish sustainable captive breeding programs [6,8].

Adequate provision of a nutritionally-balanced exogenous diet for polytrophic corals in captivity is essential to maximise survival, growth and overall fitness [9]. This can greatly reduce the time necessary to reach a size suitable for sale, transfer to an *in situ* nursery, or for transplantation onto denuded reef sites [7]. However, an optimal feeding regime for captive corals does not currently exist. Indeed, given the broad range of exogenous food sources exploited by corals, including microbiota, suspended particulate matter (SPM), and dissolved nutrients [10], an appropriate vehicle of nutrient administration is yet to be determined. Thus, an essential prerequisite for the development of optimised feeding regimes is to elucidate a readily accepted exogenous food type for captive corals.

This task is further complicated by the polytrophic nature of most hermatypic corals [6,11]. Due to the presence of phototrophic symbionts (zooxanthellae), the coral host can obtain upwards of 90% of its daily energy requirements through translocated, carbon-fixed photosynthates [11,12], making adequate zooxanthellae densities an important health determinant in corals. However, much of the material translocated to the host by the symbiont is deficient in nitrogen (N) and phosphorus (P)—critical nutrients in coral health [10,12,13]. N is a constituent of essential structural and functional macromolecules (i.e. amino acids (AA), peptides, proteins, and photosynthetic pigments) [14]. In particular, N in the form of protein plays vital roles in most biological processes, such as enzymatic catalysis, transport and storage, immunity, and growth [14]. Meanwhile, dietary P supplies phosphate required for growth and metabolism, and constitutes phospholipids, nucleic acids, and ATP [14,15]. Therefore, it is necessary for the coral host to utilise exogenous food sources to supplement its phototrophic carbon diet with materials containing adequate concentrations of N and P.

Importantly, coral heterotrophy also provides alternative forms of carbon, including lipids, their constituent classes, and fatty acids (FA) [16]. In marine ecosystems, lipids provide the densest form of energy, yielding at least one third more energy relative to proteins or carbohydrates [17]. Lipids are thus the favoured metabolic energy source in scleractinian (reef-building) corals, as evidenced by their inherently high lipid reserves constituting 10–40% ash-free dry weight (AFDW) [18].

Since energy reserves constitute a buffer against phyisco-chemical changes in the environment, this can lead to increased resistance and tolerance to stressors in corals [19]. Adequate dietary lipid is therefore a desirable characteristic in captive rearing [20], and can provide important structural and energy storage functions [21]. Specifically, structural lipids such as phospholipids are major constituents of cell membranes and intracellular organelles [15]. Storage lipids such as wax esters (WAX) and triacylglycerols (TAG) provide readily catabolised energy compounds [22]. Moreover, the FA moieties of lipids are a source of metabolic energy in the form of ATP [21], and are also required for the synthesis of new cellular lipids for growth and reproduction, turnover of existing lipids, eicosanoid production, and the regulation of membrane fluidity [21].

In accordance with the importance of these exogenous nutrients, several studies have shown the benefits of heterotrophy in corals, including increased growth, zooxanthellae density, chlorophyll *a* concentration, calcification, photosynthesis, and recovery rates following high-stress events such as bleaching and reproduction [10,23]. However, these studies are mostly limited to the addition of indiscriminate live feeds such as *Artemia* nauplii [24–27], with little consideration for the nutritional profile of these diets or the biochemical response of the coral consumer. Furthermore, although several artificial diets are commercially available for the aquarium trade, these vary widely in formulation, and are rarely assessed for efficacy (but see Forsman et al. [4]). Thus, assessing and comparing a range of dietary options, not only for their capacity to maximise growth and survival in captive corals but also to optimise overall health through comprehensive nutritional analyses is an essential prerequisite in elucidating an optimised feeding regime for *ex situ* coral rearing.

As such, this study evaluates the efficacy of a variety of heterotrophic feeding regimes for coral, including a commonly used live diet, *Artemia* nauplii (ART), a commonly used commercial diet (Reef Roids, RR), a novel, formulated diet (ATF), unfiltered seawater (RAW), and a wholly phototrophic treatment of ultra-filtered seawater (CTL). The analysis of several parameters, including growth, zooxanthellae density, and comprehensive nutritional composition were investigated in the aquaculture candidates, *Acropora millepora* and *Pocillipora acuta*, which are both common species on the Great Barrier Reef, and highly popular in home aquariums [28].

## Materials and methods

### Experimental design

Fourteen *A*. *millepora* and five *P*. *acuta* colonies were collected from Davies Reef, Queensland, Australia (lat: -18°83.162’S, long: 147°63.45’E) on the 19^th^ - 23^rd^ of July 2014 and transferred to the National Sea Simulator facility at The Australian Institute of Marine Science (AIMS, Townsville, Australia, lat: 16°17.728’S, long: 145°27.121’E). Field collections were approved by the Great Barrier Reef Marine Park Authority: G12/35236.1). Experimental units (nubbins, ~6cm) were prepared by cutting the apical branches off the colonies and gluing them onto aragonite coral plugs (Ocean Wonders). The nubbins were acclimated for four weeks by which time tissue had entirely covered the attachment area. During this period tanks were maintained in a partially re-circulated system (10L h^-1^ renewal rate), under constant temperature and light intensity of 26.8°C and 290μmol photons m^−2^s^−1^ (illumination period: 9.5h day-1, 1h ramp time), respectively. Water movement was provided by circulation pumps (Tunze, Turbelle Stream 6125).

After acclimation, three nubbins from each colony were placed into 15 x 49L tanks (24x *A*. *millepora* tank^-1^ and 15x *P*. *acuta* tank^-1^). Nubbins were maintained in ultra-filtered seawater (0.04 μm) (unfiltered in the RAW treatments) with a flow-through rate of 0.8L min^-1^ (1 turn-over h^-1^) and a circulation pump to assist with water flow (flow rate 25L min^-1^) Tunze, Turbelle Stream 6045). Temperature and light intensity remained constant at 26.8°C and 290μmol photons m^−2^s^−1^, respectively. Three replicate tanks were randomly assigned one of five feeding regimes in a single factor design:

1. *Artemia* nauplii (ART): Hatched daily. (0.05 g tank^-1^ DW).
2. Artificial (ATF): a sodium alginate-bound formulated diet developed at AIMS (Commercial in confidence formulation, details not provided). (0.05 g tank^-1^ DW).
3. Control (CTL): Flow-through ultra-filtered (0.04 μm) seawater with no additives sourced from Cleveland Bay (lat: -19°155.83’S, long: 146°88.116’E).
4. Unfiltered (RAW): Unfiltered seawater sourced from Cleveland Bay (lat: -19°155.83’S, long: 146°88.116’E) subjected to a hydro-cyclone filtration only. (Unfiltered seawater constitutes a natural source of dissolved nutrients, SPM, and microbiota, which represent potential coral food sources [29])
5. Reef Roids (RR): A commercially available aquarium diet (PolypLab, USA). (0.05 g tank^-1^ DW)

### Feeding and husbandry

The experimental duration was 90 days. Coral were fed twice daily at 1000hrs and 1600hrs for 40 minutes each time. Circulation pumps were turned off during feeding for 20 minutes to facilitate polyp capture efficiency, then turned on briefly to evoke a ‘pulse’ effect, resuspending settled feed, then left off for a further 20 minutes. Incoming water flow remained constant during feeding. Tanks were siphoned each morning to remove remaining food and debris, while twenty herbivorous snails (*Thalotia strigata*) were included in each tank to assist with algae and microfilm removal from tank walls.

Prior to experimentation, two coral nubbins per colony were sampled for initial biometry and nutritional status (T_0_). All experimental nubbins were weighed (to nearest 0.000g, Mettler-Toldeo AB204) using the buoyant weight method [30]. After 90 days, samples were analysed for buoyant weight, zooxanthellae density, proximate composition, lipid class, FA composition, and AA composition (*A*. *millepora* only).

### Zooxanthellae densities

Zooxanthellae were extracted using the tissue spraying technique described by Szmant and Gassman [31]. A 500 uL tissue slurry aliquot was taken and combined with 500 uL of 3% formalin: filtered seawater for preservation. Zooxanthellae were counted using a haemocytometer. Due to the presence of large quantities of organic material in coral skeletons [32], once the zooxanthellae aliquot was taken, the denuded skeletons were crushed and recombined with the sprayed tissue for further analyses. As such, use of the skeleton for surface area measurements was not possible, and zooxanthellae densities were standardised to AFDW.

### Proximate, lipid class, and fatty acid analysis

The tissue slurry and crushed skeletons were freeze-dried for 72h. Proximate analysis was conducted using standard procedures described by Conlan et al.[33]. Briefly, protein content was determined according to the Kjeldahl method (crude protein calculated as nitrogen × 6.25) in an automated Kjeltech (Tecator, Sweden) [34]. For total lipid content, lipids were cold extracted with dichloromethane: methanol (2:1), while ash was determined by incineration in a muffle furnace (C & L Fetlow, Model WIT, Blackburn, Victoria, Australia) at 450°C for 12h. The ash content was then subtracted from the total dry weight to obtain AFDW.

Lipid class analysis was determined using an Iatroscan MK 6s thin layer chromatography-flame ionisation detector (Mitsubishi Chemical Medience, Tokyo Japan) according to the method of Conlan et al. [33]. Briefly, each sample was spotted in duplicate on silica gel S5-chromarods (5μm particle size) with lipid separation following a two-step elution sequence: 1) elution of phosphatidylethanolamine (PE), PC, and lysophosphatidylchloline (LPC) was achieved in a dichloromethane/methanol/water (50:20:2, by volume) solvent system run to half height (~15 min); and 2) after air drying, elution of WAX, TAG, FFA, 1,2-diacylglycerol (1,2DAG), and ST was achieved in a hexane/diethyl ether/formic acid (60:15:1.5, by volume) solvent system run to full height (~30 min). Since glycolipids commonly elute with monoacylglycerols and pigments, including chlorophyll, the term “acetone mobile polar lipid” (AMPL) was used in the present study [35]. AMPL was quantified using the 1-monopalmitoyl glycerol standard (Sigma-Aldrich Co., USA), which has demonstrated a response that is intermediate between glycoglycerolipids and pigments [35].

Following initial lipid extraction, FA were esterified into methyl esters using an acid-catalysed methylation method and then analysed by gas chromatography as recently described in Conlan et al.[33].

### Amino acid analysis

For AA analysis, samples firstly underwent 24h liquid hydrolysis in 6M HCl at 110°C. As asparagine is hydrolysed to aspartic acid, and glutamine to glutamic acid, the amount of these acids is the sum of those respective components. After hydrolysis, all AA were analysed using the Waters AccQTag Ultra chemistry (Waters Acquity UPLC, Milford, USA). Samples were analysed using six replicates treatment^-1^ (two samples tank^-1^). Due to sample scarcity, only *A*. *millepora* was analysed, along with two T_0_ samples and one sample of each exogenous diet.

### Water quality analysis

Both the RAW and CTL treatments were sampled weekly for water quality analyses (Table 1). In the laboratory, the samples were analysed following standard procedures [36].

**Table 1:** Water quality analyses of CTL and RAW seawater.

Dissolved inorganic nutrients *(*μ*mol L*^*-1*^*)*	CTL	RAW
NH_4_	1.64 ± 0.43^a^	1.77 ± 0.42^a^
NO_3_	1 ± 0.14^a^	3.66 ± 3.3^a^
NO_2_	0.1 ± 0.03^b^	0.2 ± 0.11^a^
PO_4_	0.13 ± 0.02^b^	0.26 ± 0.11^a^
SiO_2_	4.68 ± 0.78^a^	5.54 ± 1.42^a^
POC *(*μ*g L*^*-1*^*)*	72.1 ± 51.7^a^	106 ± 44.6^a^
PON *(*μ*g L*^*-1*^*)*	19.1 ± 8.76^a^	23.1 ± 8.12^a^
C:N	3.23 ± 1.06^b^	4.38 ± 0.86^a^
DOC *(mg L*^*-1*^*)*	0.93 ± 0.04^a^	1.01 ± 0.14^a^
PPO_4_ *(*μ*M)*	2.55 ± 1.29^b^	11.7 ± 5.11^a^
Chl *a (*μ*g L*^*-1*^*)*	0 ± 0^b^	0.12 ± 0.06^a^
Phaeo *(*μ*g L*^*-1*^*)*	0 ± 0^b^	0.15 ± 0.07^a^
Bacteria-sized cells *(x10*^*4*^ *ml*^*-1*^*)*	28.7 ± 4.3^b^	121 ± 22.7^a^
Virus-sized cells (*x10*^*4*^ *ml*^*-1*^*)*	37.4 ± 12.7^b^	275 ± 62.6^a^

CTL = ultra-filtered (0.04 μm) seawater, RAW = unfiltered seawater. Values are presented as means ± SEM. Letters denote significant differences between treatments (P <0.05).

These analyses included five dissolved inorganic nutrients (5 DIN): NH_4_, PO_4_, NO_2_, NO_3,_ and SiO_2_, as well as particulate organic carbon (POC) and nitrogen (PON), dissolved organic carbon (DOC), particulate phosphorus (PPO_4_), and the algal pigments; chlorophyll *a* and phaeophyll. There were significantly higher amounts of PO_4_ and NO_2_ in the RAW relative to the CTL (*P*<0.05, t-test, n = 3). Levels of NH_4_, SiO_2,_ POC, PON, DOC, and PPO_4_ were also higher in the RAW; however, only PPO_4_ was significant (*P<*0.05, t-test, n = 3). The algal pigments, chlorophyll *a* and phaeophyll were present in significantly higher concentrations in the RAW (0.12 ± 0.06 and 0.15 ± 0.07 μg L^-1^ respectively), with the CTL concentrations being negligible (≤0.01 μg L^-1^). The flow cytometry results also showed significantly higher amounts of bacteria-sized cells and virus-sized particles in the RAW seawater (*P*<0.05, t-test, n = 3).

### Exogenous diet biochemical analyses

Based on dry weight, the ART diet had the highest total lipid content (225 ± 1.85 mg g sample^-1^), followed by the RR diet (184 ± 2.8 mg g sample^-1^), and the ATF diet (144 ± 2.03 mg g sample^-1^) (Table 2).

**Table 2:** Proximate (mg g sample^-1^ DW) and lipid class composition (mg g lipid^-1^) of exogenous diets.

Proximate composition	ART	ATF	RR
Lipid	225 ± 1.85	144 ± 2.03	184 ± 2.8
Protein	540 ± 1.12	198 ± 0.73	612 ± 0.72
Ash	57.1 ± 8.42	119 ± 18.4	164 ± 15.6
NFE	178 ± 3.8	539 ± 7.05	40.1 ± 6.37
**Lipid class composition**			
Wax ester	29.5 ± 5.92	42.3 ± 11.1	224 ± 2.49
Triacylglycerol	494 ± 7.06	549 ± 7.59	214 ± 1.91
Free fatty acid	132 ± 5.46	91.4 ± 1.92	75.9 ± 0.95
1,2-diacylglycerol	0 ± 0	45.5 ± 10.5	0 ± 0
Sterol	62.8 ± 4.73	41.6 ± 0.22	76.8 ± 1.73
AMPL	0 ± 0	197 ± 0.27	55.6 ± 0.03
Phosphatidylethanolamine	47.1 ± 4.54	18.2 ± 14.9	0 ± 0
Phosphatidylserine—Phosphatidylinositol	123 ± 8.71	0 ± 0	119 ± 3.39
Phosphatidylcholine	111 ± 1.38	15.3 ± 12.5	235 ± 3.61
**∑ STORAGE**	656 ± 7.53	728 ± 27.3	513 ± 1.53
**∑ STRUCTURAL**	344 ± 7.53	272 ± 27.3	487 ± 1.53
**STORAGE : STRUCTURAL**	1.91 ± 0.64	2.67 ± 3.74	1.05 ± 0.06

ART = Artemia nauplii, ATF = artificial diet, RR = Reef Roids.Values are presented as means ± SEM. NFE = Nitrogen-free extract.

Both the ART and RR diets were high in protein, constituting 540 ± 1.12 mg g sample^-1^ and 612 ± 0.72 mg g sample^-1^, respectively, while the ATF diet was comparatively low in protein (198 ± 0.73 mg g sample^-1^). Lipid class analysis revealed that both the ART and ATF diets were dominated by storage lipids (656 ± 7.53 mg g lipid^-1^ and 728 ± 27.3 mg g lipid^-1^, respectively), while the RR diet contained similar amounts of storage and structural lipids (513 ± 1.53 mg g sample^-1^ and 487 ± 1.53 mg g sample^-1^, respectively) (Table 2). Notable differences in the diet FA compositions included markedly higher 18:3n-3 in the ART treatment (ART: 152 ± 2.43 mg g lipid^-1^ vs ATF: 1.47 ± 0.7 mg g lipid^-1^ and RR: 5.33 ± 0.23 mg g lipid^-1^) (Table 3).

**Table 3:** Fatty acid (mg g lipid^-1^) and amino acid (mg g sample^-1^ DW) composition of exogenous diets.

Fatty acids	ART	ATF	RR
10:0	0.24 ± 0.08	0.34 ± 0.24	0.05 ± 0
14:0	2.29 ± 1.87	40.1 ± 9.87	40.9 ± 2.02
16:0	65.8 ± 1.43	160 ± 37.7	86.3 ± 4.34
18:0	19.8 ± 0.81	35 ± 6.97	4.63 ± 0.25
**∑ SFA**	93.6 ± 1.78	247 ± 58	134 ± 6.85
18:1n-9	112 ± 2.2	35.2 ± 7.26	43.3 ± 2.14
16:0-OH	0.17 ± 0	0 ± 0	1.26 ± 0.04
**∑ MUFA**	176 ± 3.53	49.9 ± 10.6	96.6 ± 5.07
18:3n-3	152 ± 2.43	1.47 ± 0.7	5.33 ± 0.23
18:4n-3	18 ± 0.01	0 ± 0	11.3 ± 0.41
20:4n-3	0 ± 0	1.48 ± 0.62	1.87 ± 0.13
20:5n-3	7.77 ± 0.02	5.6 ± 1.1	38.3 ± 1.79
22:5n-3	0 ± 0	1.16 ± 0.29	0.69 ± 0.02
22:6n-3	0 ± 0	81 ± 19.5	18.8 ± 0.89
18:2n-6	35.5 ± 0.55	6.16 ± 4.46	0.08 ± 0.01
18:3n-6	2.12 ± 0.01	0.29 ± 0.24	0.56 ± 0.01
20:3n-6	0.36 ± 0.2	1.39 ± 0.5	0.2 ± 0
20:4n-6	2.37 ± 0.12	4.95 ± 1.06	0.01 ± 0
22:4n-6	0.18 ± 0.02	0 ± 0	0.21 ± 0.01
**∑ PUFA**	221 ± 3.61	137 ± 35.7	79 ± 3.39
**TOTAL**	493 ± 10.9	436 ± 105	323 ± 15.9
**∑ n-3 PUFA**	178 ± 2.48	96.7 ± 23.1	76.8 ± 3.46
**∑ n-6 PUFA**	41.5 ± 0.9	40.3 ± 12.6	1.87 ± 0.06
**∑ n-3 LC-PUFA**	8.71 ± 0.07	95.3 ± 23.8	60.2 ± 2.82
**∑ n-6 LC-PUFA**	2.98 ± 0.35	33.7 ± 8.37	0.67 ± 0.07
**Amino acids**			
Histidine	11.8	2.2	12.3
Serine	27.5	4.2	24.2
Arginine	35.2	6.4	35.0
Glycine	24.8	6.2	28.2
Aspartic acid	37.8	8.9	46.9
Glutamic acid	57.1	12.7	61.0
Threonine	23.4	4.1	26.3
Alanine	24.2	5.9	26.7
Proline	25.7	4.9	22.1
Lysine	37.6	6.8	34.4
Tyrosine	15.9	2.4	22.4
Methionine	12.2	2.3	17.0
Valine	27.3	5.2	31.7
Isoleucine	24.3	4.1	29.6
Leucine	36.4	6.7	44.1
Phenylalanine	22.3	3.6	28.3
**TOTAL**	444	86.7	490

ART = Artemia nauplii, ATF = artificial diet, RR = Reef Roids. Values are presented as means ± SEM (where available).

Notably, higher DHA concentrations were evident in the ATF diet (ATF: 81 ± 19.5 mg g lipid^-1^ vs ART: 0 ± 0 mg g lipid^-1^ and RR: 18.8 ± 0.89 mg g lipid^-1^), while the RR diet was low in n-6 PUFA (RR: 1.87 ± 0.06 mg g lipid^-1^ vs ART: 41.5 ± 0.9 mg g lipid^-1^ and ATF: 40.3 ± 12.6 mg g lipid^-1^).

The ART and RR diets were fairly similar in AA composition (444 mg g sample^-1^ and 490 mg g sample^-1^, respectively), while the ATF diet contained markedly less (86.7 mg g sample^-1^) (Table 3).

### Statistical analysis

Statistical analysis for all data was implemented in R software version 2.3.1 [37]. Normality and heteroscedasticity were determined by performing the Shapiro-Wilk and Bartlett’s tests, respectively. Due to heteroscedasticity, data were transformed using log_10_(χ+1). Differences between treatments were then analysed using a linear mixed effects model (*lme4* package, [38]) where Treatment was included as a fixed effect, and Tank was included as a random effect [39]. Where significant differences were detected, a Tukey’s *post hoc* test was employed at a significance level of P<0.05 to determine which groups differed (*multcomp* package [40]). The statistical effect of different genotypes was analysed with a two-way ANOVA at a significance level of P<0.05. Water quality results of the CTL and RAW treatments were analysed using a *t*-test. The datasets generated during the current study are available from the corresponding author on reasonable request.

## Results

Consumption of all particulate diets was observed visually in both coral species by tentacle capture and retraction into the polyp. Nubbin mortality during this study was negligible (<5%) and no significant differences were detected between genotypes for the biometrics described below (P>0.05). These parameters will therefore not be further considered.

### Growth

**Fig 1:**
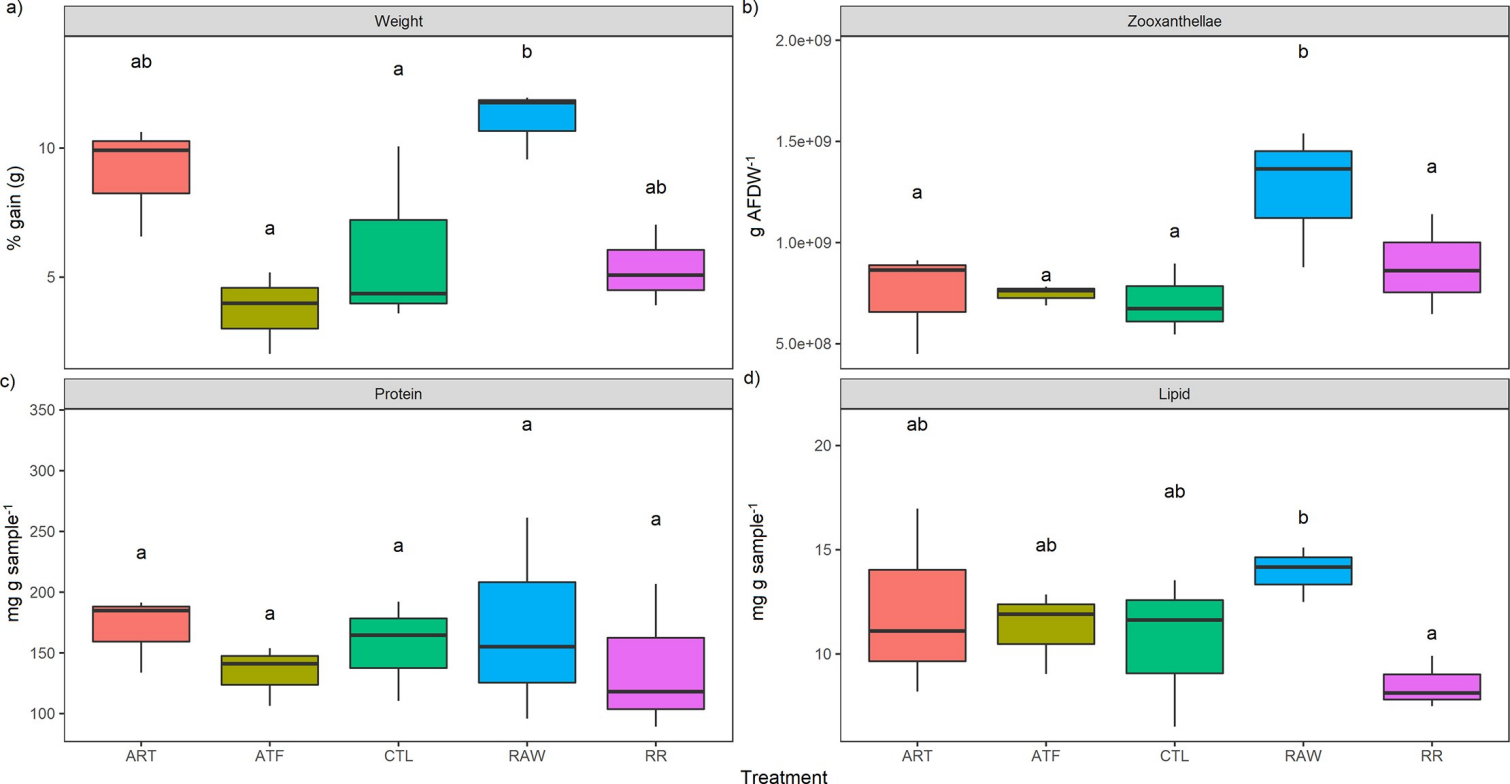
**Effect of five different feeding regimes on the a) growth, b) zooxanthellae density, c) total protein, and d) total lipid concentration of *Acropora millepora* after 90 days.** Boxes encompass the 25^th^ and 75^th^ percentile, dots denote single outliers. n = 3. Boxes in the same plot that do not share the same letters are significantly different (*P*<0.05). ART = *Artemia* nauplii, ATF = artificial diet, CTL = ultra-filtered (0.04 μm) seawater, RAW = unfiltered seawater, RR = Reef Roids.

**Fig 2:**
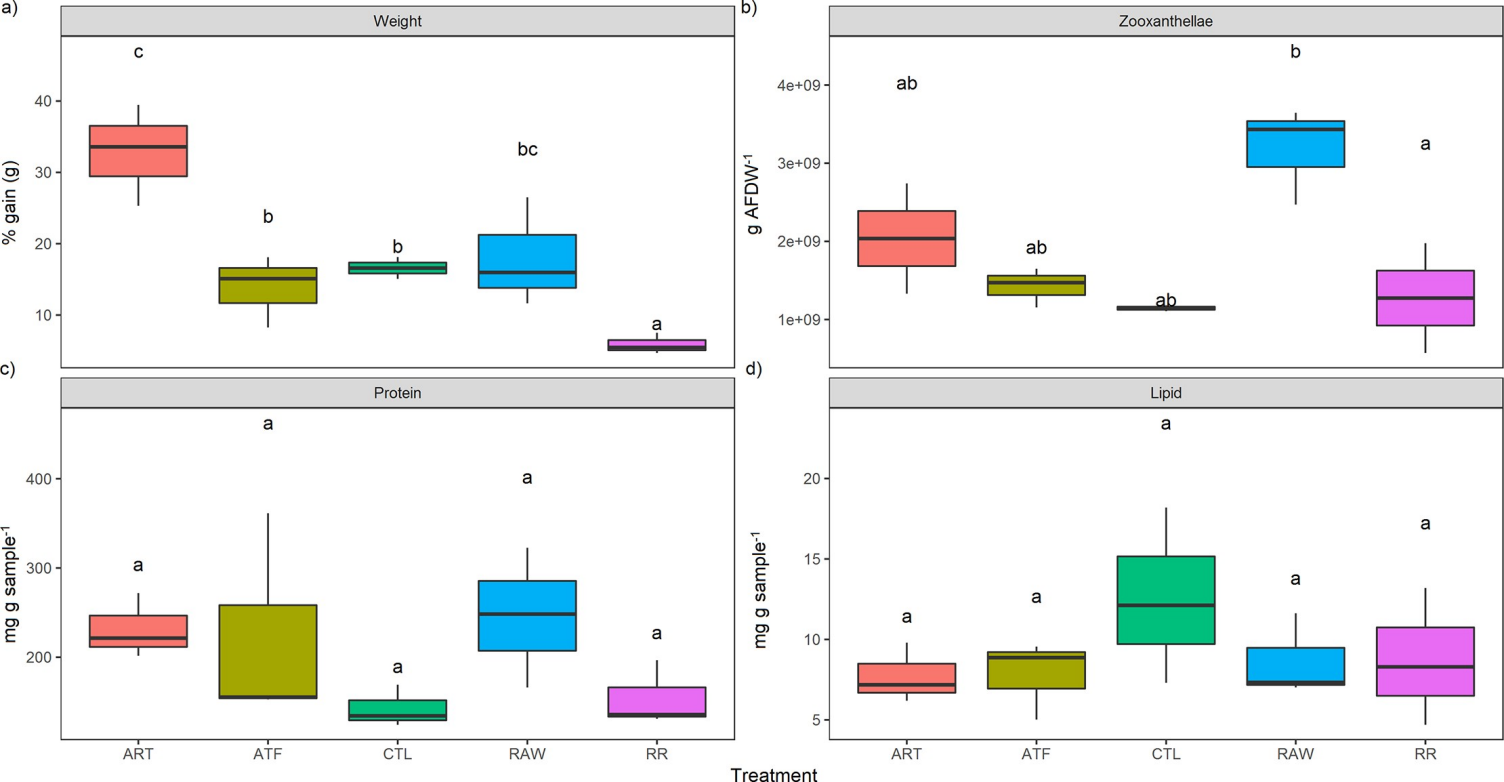
**Effect of five different feeding regimes on the a) growth, b) zooxanthellae density, c) total protein and, d) total lipid concentration of *Pocillopora acuta* after 90 days.** Boxes encompass the 25^th^ and 75^th^ percentile, dots denote single outliers. n = 3. Boxes in the same plot that do not share the same letters are significantly different (*P*<0.05). ART = Artemia nauplii, ATF = artificial diet, CTL = ultra-filtered (0.04 μm) seawater, RAW = unfiltered seawater, RR = Reef Roids.

Corals in all treatments grew over the course of the experiment. After 90 days, one treatment stood out as clearly superior for each species. For *A*. *millepora*, this was in the RAW treatment, exhibiting significant weight gain compared to the CTL and ATF treatments (11.1 ± 2.19%) (P<0.05) (mean ± SEM, Fig 1A). For *P*. *acuta*, this was in the ART treatment (32.8 ± 6.74%), which was significantly greater compared to all other treatments except the RAW (P<0.05). Likewise, one treatment also exhibited considerably lower weight gain in both species. For *A*. *millepora* this was the ATF treatment (3.72 ± 1.99%) (Fig 1A), while for *P*. *acuta* this was the RR treatment (5.89 ± 3.05%) (P<0.05) (Fig 2A).

### Zooxanthellae density

For *A*. *millepora*, the RAW treatment exhibited zooxanthellae densities that were an order of magnitude higher than all other treatments (P<0.05) (Fig 1B). The RAW treatment also recorded the highest zooxanthellae density for *P*. *acuta*, and this was significantly greater than the RR treatment (Fig 2B).

### Proximate composition

Feeding regime had no significant effect on total protein for *A*. *millepora* (~9–13 mg g sample^-1^) (Fig 1C). Protein levels in *P*. *acuta* were markedly higher in the ART and RAW treatments (~11 mg g sample^-1^), which were almost two-fold higher than all other treatments (~6 mg g sample^-1^), however this was not significant (Fig 2C). The RAW treatment culminated in the highest total lipid for *A*. *millepora* (~14 mg g sample^-1^), and this was significantly higher compared to the RR treatment (P<0.05) (Fig 1D). Meanwhile, the highest total lipid for *P*. *acuta* was recorded in the CTL treatment (~13 mg g sample^-1^), although this was not significantly different to the other treatments (7.9–8.3 mg g sample^-1^) (Fig 2D).

### Amino acid composition of *A*. *millepora*

The total AA concentration of the RAW treatment (~6090 μg g sample^-1^) was significantly higher than all other treatments (~4180–4440 μg g sample^-1^) with the exception of the RR treatment (~4930 μg g sample^-1^) (Table B in S1 Supporting Information). All treatments were dominated by glutamic acid (~17%), while histidine and methionine generally showed the lowest abundance (<2%). Generally, the RAW treatment resulted in significantly higher amounts of most individual AA compared to the ART, ATF, and CTL treatments (P<0.05). Interestingly, the only significant difference between the RAW and RR treatments was in methionine (P<0.05). Despite the large quantitative increases in the RAW treatment, alanine was the only AA that was significantly higher on a qualitative basis), and this was only compared to the ATF and CTL treatments.

### Lipid class composition

**Fig 3:**
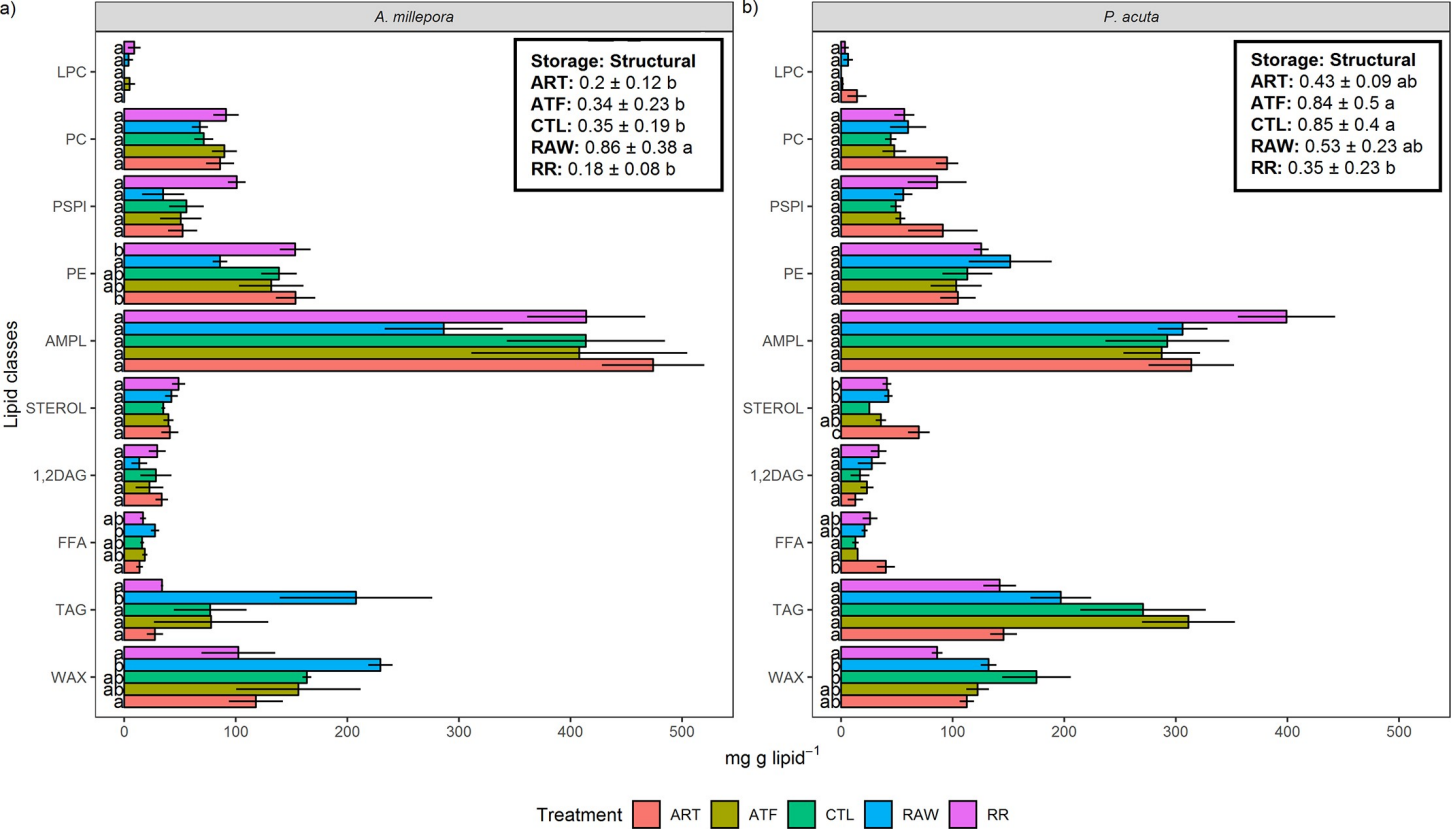
**Effect of five different feeding regimes on the lipid class composition of a) *Acropora millepora* and b) *Pocillopora acuta* after 90 days (mg g lipid**^**-1**^**).** WAX = wax ester, TAG = triacylglycerol, FFA = free fatty acids, 1,2DAG = 1,2diacylglycerol, ST = sterol, AMPL = acetone-mobile-polar-lipid, PE = phosphatidylethanolamine, PS-PI = phosphatidylserine-phosphatidylinositol, PC = phosphatidylcholine. Values are presented as means ± SEM. n = 3. Letters denote significant differences between treatments for each lipid class (*P* <0.05). ART = *Artemia* nauplii, ATF = artificial diet, CTL = ultra-filtered (0.04 μm) seawater, RAW = unfiltered seawater, RR = Reef Roids.

For *A*. *millepora*, the RAW treatment resulted in markedly greater levels of lipid classes associated with storage: WAX and TAG, which were significantly higher than in the ART and RR treatments for WAX, and all treatments for TAG (P<0.05) (Fig 3A). The RAW treatment also resulted in the lowest levels of acetone mobile polar lipid (AMPL). Correspondingly, the RAW treatment showed a significantly higher storage:structural lipid ratio (0.86 ± 0.38) compared to all other treatments (~0.18–0.35).

In *P*. *acuta*, WAX concentrations in the RR treatment (~86 mg g lipid^-1^) were significantly lower than the CTL and RAW treatments (135–175 mg g lipid^-1^) (Fig 3B). Additionally, TAG concentrations were greatest in the ATF treatment (~317 mg g lipid^-1^), although this was only significant compared to the other treatments. The ART treatment exhibited the highest concentration of several lipid classes, including free fatty acids (FFA), ST, phosphatidylserine- phosphatidylinositol (PS-PI), and phosphatidylcholine (PC). The ATF and CTL treatments recorded the highest storage:structural lipid ratio for this species (~0.85), while the RR was lowest (~0.35).

### FA composition

**Fig 4:**
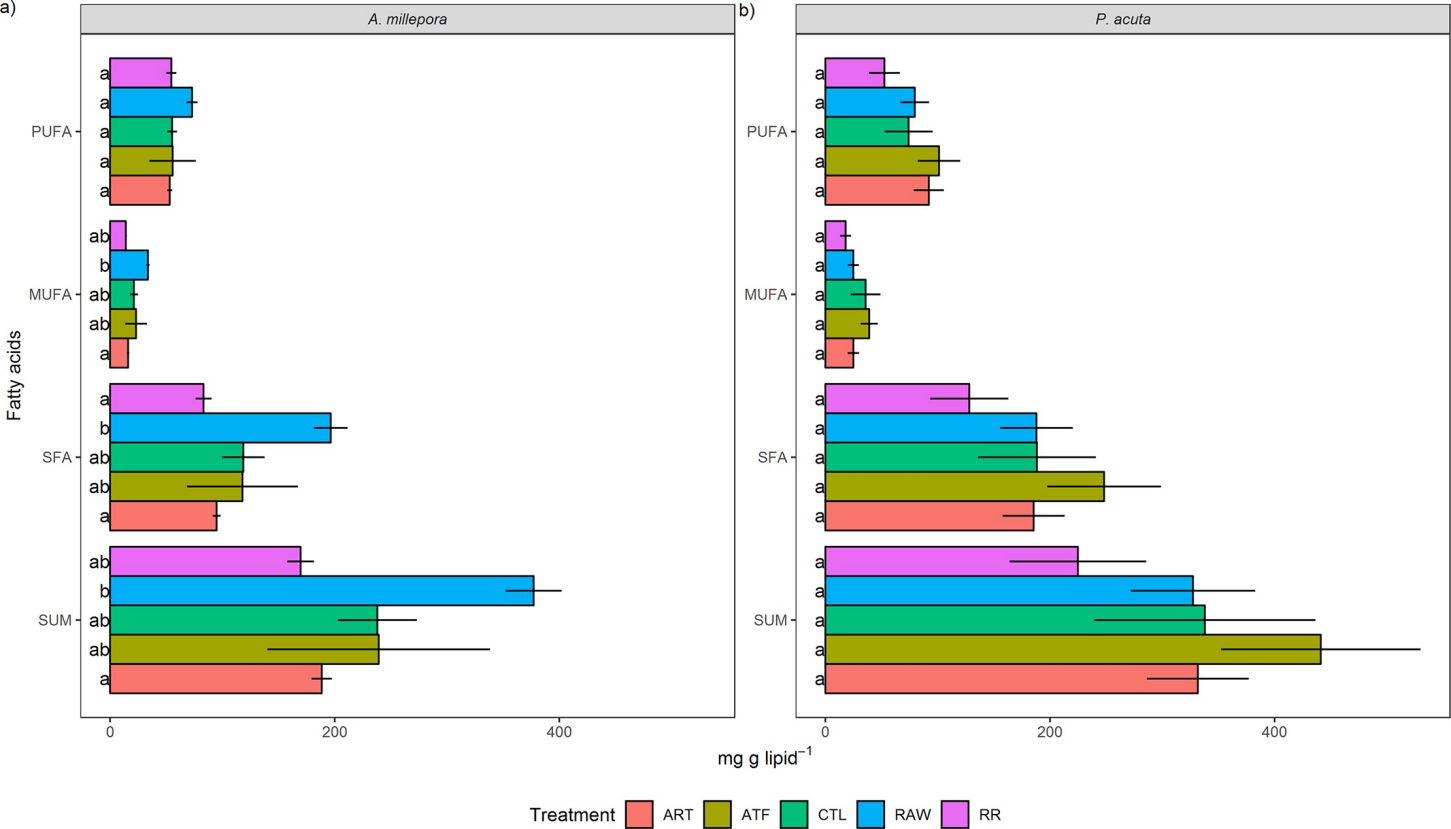
**Effect of five different feeding regimes on the major fatty acid class composition of a) *Acropora millepora* and b) *Pocillopora acuta* after 90 days (mg g lipid**^**-1**^**).** SFA = saturated fatty acids, MUFA = monounsaturated fatty acids, PUFA = polyunsaturated fatty acids. Values are presented as means ± SEM. n = 3. Letters denote significant differences between treatments for each fatty acid class (*P* <0.05). ART = *Artemia* nauplii, ATF = artificial diet, CTL = ultra-filtered (0.04 μm) seawater, RAW = unfiltered seawater, RR = Reef Roids.

The total FA concentration was significantly higher in the RAW treatment for *A*. *millepora* (~373 mg g lipid^-1^) compared to the ART treatment (183 mg g lipid^-1^) (Fig 4A). Correspondingly, the RAW treatment also exhibited markedly higher quantitative amounts of saturated fatty acids (SFA), monounsaturated fatty acids (MUFA), and polyunsaturated fatty acids (PUFA). Meanwhile, *P*. *acuta* recorded the highest FA concentration in the ATF treatment (~441 mg g lipid^-1^), applying also to the total concentrations of SFA, MUFA, and PUFA (Fig 4B).

**Fig 5:**
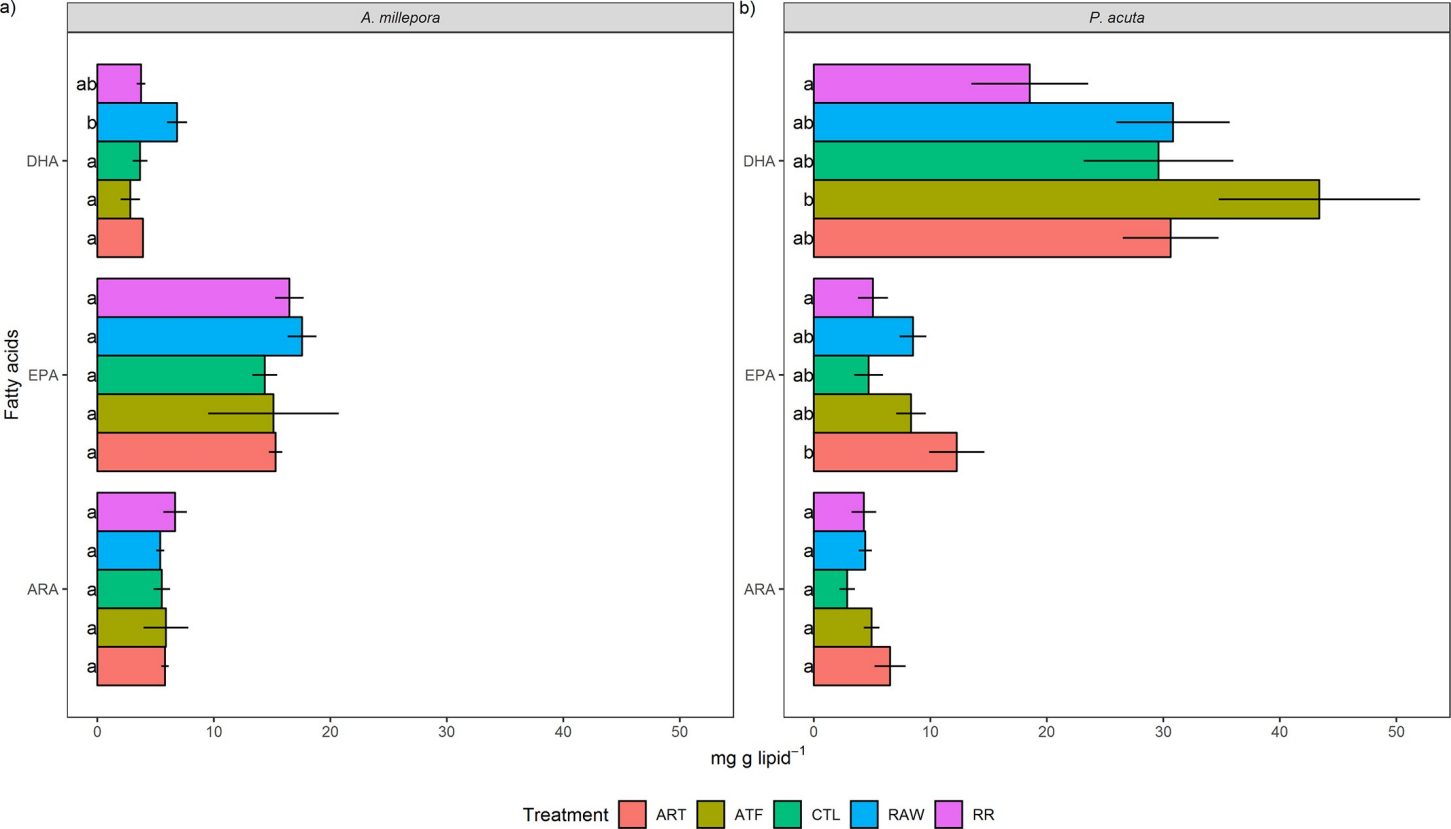
**Effect of five different feeding regimes on the major long-chain polyunsaturated fatty acid composition of a) *Acropora millepora* and b) *Pocillopora acuta* after 90 days (mg g lipid**^**-1**^**).** ARA = arachidonic acid (20:4n-6), EPA = eicosapentaenoic acid (20:5n-3), DHA = docosahexaenoic acid (22:6n-3). Values are presented as means ± SEM. n = 3. Letters denote significant differences between treatments for each fatty acid (*P* <0.05). ART = *Artemia* nauplii, ATF = artificial diet, CTL = ultra-filtered (0.04 μm) seawater, RAW = unfiltered seawater, RR = Reef Roids.

Quantitatively, for *A*. *millepora*, most individual FA in the RAW treatment were significantly higher compared to the ART and RR treatments, the most noteworthy being 14:0, 16:0, 18:1n-9, 16:0-OH, 22:6n-3 (DHA), and 18:3n-6 (mg g lipid^-1^) (Fig 5A, Table D in S1 Supporting Information). Meanwhile, the ATF treatment recorded the highest DHA content in *P*. *acuta* (~44 mg g lipid^-1^), and this was significant compared to the RR treatment (~18 mg g lipid^-1^) (Fig 5B, Table E in S1 Supporting Information). Additionally, the ART treatment contained significantly higher concentrations of 20:5n-3 (EPA) compared to the RR treatment for this species (P<0.05).

## Discussion

The present study found large variation in the growth and nutritional composition of two scleractinian coral species subjected to five feeding regimes in captivity. These results are important since an optimised heterotrophic diet can improve the overall health of captive corals, maximising stress resistance and vigour, and advancing husbandry techniques for commercial and conservation benefits [3,41].

After 90 days, both species demonstrated significantly greater growth in one treatment (*A*. *millepora*–RAW, *P*. *acuta*–ART), and a comparatively poor growth response to another (*A*. *millepora*–ATF, *P*. *acuta*–RR). The growth rates of *A*. *millepora* in the RAW treatment (~0.12% increase in weight day^-1^ (g)) were comparable to those previously recorded for this species under similar conditions (~0.14% day^-1^) [42]. These results also mirror those recorded in *Acropora* recruits [43], whereby growth was significantly greater in the RAW treatment compared to the ATF. This indicates that the requirements for key characteristics in heterotrophic diets remain similar for *A*. *millepora* from benthic settlement through to adulthood.

With regard to *P*. *acuta*, the ART treatment recorded the highest growth, and this was two-fold greater than the next highest growth, which was in the RAW treatment. This agrees well with the results of Osinga et al. [44], who showed that *Pocillopora damicornis* supplied with *Artemia* nauplii grew to twice the weight of those supplied with unfiltered seawater only. In another similar study, Forsman et al. [4] found Reef Roids to elicit the highest growth in *P*. *damicornis* compared to several other commercially available artificial foods (not tested here) after three months. However, this weight gain only amounted to ~7.5%, which is only ~1.6% higher than that achieved for the RR treatment over the same period in the present study. Thus, while Reef Roids may be superior to other common aquarium diets, it is clearly not an optimal feeding option, with the ART treatment proving capable of supporting five-fold greater growth for *P*. *acuta*. Since colony size is an important parameter for coral culture, capitalisation on heterotrophy via the identification of an optimal diet can greatly reduce the time for corals to reach a suitable size for use in reef restoration efforts or the aquarium trade [7].

The increased growth in the ART and RAW treatments compared to the CTL likely reflects the assimilation of the growth-promoting nutrients, organic N, organic P, and inorganic minerals(Tables 1 & 2), considering photosynthates are generally deficient in these [10,44] and *Artemia* has previously been suggested to constitute a significant source of organic P for coral [45]. Furthermore, since zooxanthellae are limited by N and P *in hospite* [44], these nutrients can also increase zooxanthellae proliferation, pigment production, and photosynthesis rates in corals [10,46]. Correspondingly, the treatments exhibiting high growth also recorded significantly higher zooxanthellae densities (*A*. *millepora*–RAW, *P*. *acuta*–ART and RAW). However, despite exhibiting the second highest growth in *A*. *millepora*, the zooxanthellae density in the ART treatment was considerably lower than the RAW. Likewise, despite the superior growth of *P*. *acuta* in the ART treatment, its zooxanthellae density was surpassed by the RAW. This may reflect this species’ rapid growth in the ART treatment, creating a lag-effect in zooxanthellae populations, as rapidly forming tissue takes time to be completely colonised [47].

Tellingly, the treatments that elicited the greatest growth for both species (ART and RAW) were live or ‘natural’, while the poor performing diets were artificial and inert (ATF and RR). Introducing an artificial diet to a captive species is traditionally problematic [21]. In particular, digestibility is a pertinent issue for corals given their unspecialised digestive systems [27]. Considering this, the alginate used to bind the ATF diet may have impeded its digestibility, as has been shown in other unspecialised digestive systems such as those of larval fishes [48]. Thus, the ATF diet may be better delivered in a dissolved format, as dissolved nutrients are a known food source in corals [10]. Indeed, water quality analyses showed significantly higher dissolved N and P levels in the RAW treatment compared to the CTL. Slightly elevated N and P concentrations have shown increased growth and photosynthetic efficiency in corals [49], since they can be assimilated by zooxanthellae and transported to the host for incorporation into vital molecules including DNA, RNA, proteins, and phospholipids [50].

Furthermore, there are several benefits of live or natural diets compared to artificial diets. These include feeding stimulation and attraction through movement, enzymatic activity that aids digestion, palatability, as well as appropriate nutritional format or composition [48,51]. Although the absence of these factors may have resulted in low consumption of the ATF and RR diets, it is apparent that the amount consumed negatively impacted growth, zooxanthellae densities, and total protein and lipid concentrations. Indeed, the twice-daily meal format of the ATF and RR diets may have caused nutritional overloading of the coral digestive system, which is a recognised problem with formulated feeds [52].

Unlike *A*. *millepora*, *P*. *acuta* recorded superior weight gain in the ART treatment, indicating this species obtained sufficient energy during the two daily meals to supersede the RAW treatment. This may be due, firstly, to the ART treatment’s nutritional composition, which may be better suited to the metabolic requirements of this species (discussed below). Secondly, greater consumption, since *Pocillopora sp*. have shown significantly higher capture rates of *Artemia* nauplii compared to *Acropora sp*. [53,54]. Thirdly, this species may have a preference for larger, motile prey. Indeed, *P*. *damicornis* has been shown to more readily capture *Artemia* nauplii compared to SPM [55], which represents a key attribute of the RAW treatment.

Furthermore, Lewis and Price [56] noted that pocilloporids only employed tentacular feeding, whereas acroporids employed both tentacular and mucus feeding. Thus, in addition to dissolved nutrients, other properties of the RAW treatment, such as SPM and phytoplankton, may be better suited to *A*. *millepora* due to a preference for smaller, less motile prey. Indeed, coral feeding on microalgae has recently been shown to be more prevalent in scleractinian corals than previously suspected [57]. Moreover, Wijgerde and Laterveer [58] recorded growth rates up to eight-fold higher than the present study in *A*. *millepora* fed a phytoplankton mixture thrice weekly. The presence of phytoplankton in the RAW treatment is supported by the flow cytometry, chlorophyll, and phaeophyll results, which were significantly higher compared to the CTL. Phytoplankton consumption has also been shown in pocilloporids [9], which may also explain the positive response to the RAW treatment for *P*. *acuta*. Regardless, these results highlight the need for diets tailored to different coral genera to achieve maximum growth.

The rapid growth of *P*. *acuta* in the ART treatment was reflected in the total protein concentration. High protein levels are characteristic of actively growing sites [59], where protein synthesis and retention facilitates calcification and tissue synthesis [60]. Proteins and AA are continually used by animals to build new proteins (e.g. growth) or to replace existing proteins (e.g. maintenance), necessitating their regular intake [61]. Conversely, inadequate dietary protein results in reduced growth due to protein withdrawal from less vital tissues to maintain functioning in more vital tissues [61].

There were no significant differences in the total protein concentrations for *A*. *millepora* across the five treatments, despite the significantly higher growth in the RAW treatment. However, the RAW did contain significantly higher total AA levels compared to all other treatments, including most individual AA (μg g sample^-1^). This suggests increased non-protein N in the ART, ATF, CTL, and RR treatments compared to the RAW, possibly reflecting increased concentrations of pigments, inorganic N, or chitin in these treatments [62]. Higher total AA concentrations in the RAW treatment may also reflect the increased zooxanthellae density. This is supported by significantly higher alanine proportions in the RAW treatment compared to the ATF and CTL (% AA). Alanine has been identified as the principle AA translocated from zooxanthellae to host in invertebrate symbioses [63].

The overall AA composition of *A*. *millepora* was consistent with other Scleractinians [63,64], with the major AA being glutamic acid, aspartic acid, and glycine. Glutamic acid contributed the majority of the total AA, likely because this is the synthesis precursor of all other AA [14]. Meanwhile, histidine was present in the lowest concentrations, possibly reflecting its comparatively complex formation process [14], or a low physiological requirement.

In *A*. *millepora*, the growth and zooxanthellae densities obtained in the RAW treatment were mirrored in the total lipid concentration (~14 mg g sample^-1^), recording significantly higher levels compared to most treatments (~8.8–11.3 mg g sample^-1^). The lipid content in *A*. *millepora* from Davies Reef during a similar time of year was ~12.3–29.9 mg g sample^-1^ (Conlan et al. unpublished data), suggesting the lipid content was increased in the RAW treatment and reduced in the others. The lipid class results support this, since the reduced lipid concentration in the ART, ATF, CTL, and RR treatments for *A*. *millepora* was largely attributable to significantly lower WAX and TAG concentrations. These classes serve as large and important energy reserves in healthy corals [22], and low TAG in particular, can signify stress, given its catabolism for energy [65,66].

All treatments with the exception of RAW were dominated by AMPL, constituting almost half the total lipid concentration. Lipids play mitigating roles during nutrient deprivation in plants [67], and replacement of membrane phospholipids with non-phosphorus glycerolipids (a major AMPL constituent) promotes P remobilisation [68]. This is a typical metabolic signature associated with lipid remodeling during P deprivation in plants [68], and may thus apply to the zooxanthellae populations in the present study, since the CTL seawater contained half the dissolved P concentration of the RAW seawater.

The total FA concentration for *A*. *millepora* in the RAW treatment mirrored the high TAG concentration, since TAG contains three esterified FA, while phospholipids, ST, and AMPL possess two or less [69]. Furthermore, SFA and MUFA are mainly stored as WAX and TAG in corals [65], and these groups were correspondingly found in the highest concentrations in the RAW treatment. SFA and MUFA also represent readily catabolised energy sources [70], again demonstrating the increased energy reserves in this treatment.

Notably, EPA and 20:4n-6 (ARA) were not significantly different between treatments in *A*. *millepora*. These FA are known eicosanoid precursors, which are critical for numerous physiological processes, including pigmentation and immune function [21], suggesting selective retention due to their indispensable nature, host biosynthesis, or zooxanthellate origin [71]. Preferentially sparing certain FA during lipid catabolism reflects a biochemical strategy to preserve the most essential components of biological membranes during stress [72]. In contrast, DHA was significantly higher in the RAW treatment, demonstrating its importance in improving *A*. *millepora* health. Whether this was sourced from heterotrophic input or increased *de novo* synthesis attributable to higher zooxanthellae densities is uncertain. Indeed, the presence of zooxanthellae and other endogenous algae and bacteria within corals shrouds the origin of PUFA, as the overall composition may reflect nutrients derived from multiple sources [73]. This is particularly relevant when considering some zooxanthellae clades are able to synthesise LC-PUFA [71].

The lipid results were strikingly different for *P*. *acuta* in comparison to *A*. *millepora*, with total lipid concentrations being significantly higher in the CTL and all other treatments indistinguishable. Increased lipid in the CTL treatment, coupled with low protein, indicates N deprivation [74]. Photosynthates derived from N-limited zooxanthellae have been termed ‘junk food’ for coral, since they only provide the host with carbon-rich metabolic energy, not the N-rich compounds needed for framework maintenance and biosynthesis [44]. Furthermore, heterotrophic nutrients are generally directed toward growth and symbiont proliferation rather than accumulated lipid reserves, as the coral’s energetic requirement is largely met by phototrophy [8]. This agrees with the low growth and zooxanthellae densities in the ATF and RR treatments for both species, yet no major differences in their lipid concentration.

Interestingly, although the best performing treatment for *A*. *millepora*, RAW, exhibited high TAG, SFA, and DHA levels, this species responded poorly to the ATF diet, which was abundant in these specific nutrients, again suggesting it was delivered in an inappropriate nutritional format, or consumption was low. In contrast, the ATF dietary profile was largely reflected in *P*. *acuta*, which exhibited high TAG, SFA, and DHA concentrations, demonstrating efficient assimilation. However, this did not translate into superior performance, suggesting its nutritional composition was ill-suited to this species’ dietary requirements.

Developing an artificial diet for corals presents many advantages, including nutrient optimisation, consistent quality, and reduced rearing costs [4]. However, elucidating optimal diets for new species requires numerous incremental steps to establish basal dietary information. Since the quantitative requirements for crude nutrients are not known in corals, an initial broad scale approach is required to determine an optimal vehicle of nutrient delivery. Thus, in the absence of more definitive work, the ATF diet in the present study was formulated based on the nutritional composition of wild-caught corals (Conlan et al., unpublished data), since lipid class and FA profiles, in particular, can closely reflect qualitative requirements and natural diet composition [75]. Consequently, the ATF diet contained a larger neutral lipid proportion compared to the ART and RR diets. However, in unspecialised digestive systems such as those of larval fishes, there appears to be a greater capacity to digest dietary lipids in the phospholipid form, given the high polarity of neutral lipids, such as TAG [21]. Dietary lipid is also an important source of essential fatty acids (EFA), yet not all lipid classes are equally effective in delivering EFA, and phospholipids tend to be a richer source than neutral lipids [76]. Furthermore, given the capacity of zooxanthellae to synthesise some LC-PUFA [71,73], their inclusion may be superfluous in exogenous coral diets. Therefore, future studies should examine diets richer in both phospholipids and short-chain FA.

The present study indicates that *Artemia* nauplii can serve as a suitable feeding regime for captive *P*. *acuta*. Future investigations should seek to optimise this through manipulation of feed density and frequency. In contrast, the results suggest considerable work is still required to optimise feeding regimes for captive *A*. *millepora* to achieve growth and health levels akin to those possible with natural feeding regimes. Despite the superiority of the RAW treatment, unfiltered seawater does not present a feasible option for coral culture due to potential bacteria and pathogen introduction, as well as seasonal fluctuations in salinity and nutrient loads. However, these results do provide important insight into the dietary requirements of *A*. *millepora*, and future investigations should examine the RAW treatment’s beneficial characteristics, including dissolved nutrients and phytoplankton, to progress toward an optimal feeding regime for this species.

## Supporting information

S1 Supporting information Supplementary tables A—E.
(DOC)
