# Supplementary material for: Comparing the capacity of five different dietary treatments to optimise growth and nutritional composition in two scleractinian corals
Source: PLoS One. 2018 Nov 28;13(11):e0207956. doi: 10.1371/journal.pone.0207956 (PMC6261599; doi:10.1371/journal.pone.0207956)
Supplement: S1 Supporting information — Supplementary tables A—E. (DOC) [file pone.0207956.s001.doc]

# **Table A Proximate (mg g sample^-1^) and lipid class composition (mg g lipid^-1^) of initial samples of *Acropora millepora* and *Pocillopora acuta*.** Values are presented as means ± SEM.

| **Proximate composition** | ***A. millepora*** | ***P. acuta*** |
| --- | --- | --- |
| Lipid | 11.6 ± 3.24 | 9.54 ± 2.54 |
| Protein | 20.8 ± 4.91 | 17.7 ± 6.44 |
| Ash | 920 ± 11.8 | 952 ± 5.57 |
| **Lipid class composition** | | |
| Wax ester | 48.9 ± 28.3 | 102 ± 37.7 |
| Triacylglycerol | 29.4 ± 10.6 | 121 ± 43.7 |
| Free fatty acid | 14.7 ± 5.07 | 13.4 ± 2.97 |
| 1,2-diacylglycerol | 29.4 ± 19.7 | 41 ± 8 |
| Sterol | 44.4 ± 9.17 | 29.5 ± 4.8 |
| AMPL | 471 ± 138 | 419 ± 123 |
| Phosphatidylethanolamine | 168 ± 76.7 | 164 ± 14.8 |
| Phosphatidylserine - Phosphatidylinositol | 110 ± 85.3 | 40.8 ± 33.4 |
| Phosphatidylcholine | 74.9 ± 17.9 | 50.9 ± 6.57 |
| Lysophosphatidylcholine | 9.01 ± 11 | 18 ± 15.2 |
| **∑STORAGE** | 122 ± 60.3 | 278 ± 90.6 |
| **∑STRUCTURAL** | 878 ± 60.3 | 722 ± 90.6 |
| **STORAGE:STRUCTURAL** | 0.15 ± 0.08 | 0.42 ± 0.2 |

# **Table B Effect of five different feeding regimes on the amino acid composition of *Acropora millepora* and *Pocillopora acuta* after 90 days (µg g sample^-1^).** Values are presented as means ± SEM. Letters denote significant differences between treatments for each amino acid (P <0.05). ART = *Artemia* nauplii, ATF = artificial diet, CTL = ultra-filtered (0.04 μm) seawater, RAW = unfiltered seawater, RR = Reef Roids.

| **Amino acids** | **T_0_** | **ART** | **ATF** | **CTL** | **RAW** | **RR** |
| --- | --- | --- | --- | --- | --- | --- |
| Histidine | 131 ± 8.74 | 77.1 ± 6.54^b^ | 76.2 ± 16.6^b^ | 84.5 ± 4.21^b^ | 121 ± 6.66^a^ | 96.8 ± 12.7^ab^ |
| Serine | 384 ± 20.1 | 249 ± 13.7^b^ | 219 ± 40.3^b^ | 261 ± 11.8^b^ | 362 ± 12.8^a^ | 295 ± 33.8^ab^ |
| Arginine | 387 ± 22.4 | 263 ± 25.9^b^ | 237 ± 45.2^b^ | 295 ± 15.9^ab^ | 376 ± 20.9^a^ | 310 ± 31.5^ab^ |
| Glycine | 444 ± 23 | 262 ± 18.6^b^ | 246 ± 30.7^b^ | 271 ± 11.9^b^ | 383 ± 8.97^a^ | 308 ± 36.1^ab^ |
| Aspartic acid | 766 ± 40.6 | 547 ± 38.4^b^ | 476 ± 70.5^b^ | 571 ± 16.8^b^ | 742 ± 32.7^a^ | 615 ± 63.1^ab^ |
| Glutamic acid | 1170 ± 63.3 | 716 ± 40.4^b^ | 589 ± 126^b^ | 753 ± 32.5^b^ | 1080 ± 47.7^a^ | 852 ± 101^ab^ |
| Threonine | 328 ± 17.8 | 213 ± 12.3^b^ | 186 ± 36.6^b^ | 228 ± 11.4^b^ | 311 ± 12.7^a^ | 256 ± 27.2^ab^ |
| Alanine | 325 ± 19.5 | 208 ± 11.9^b^ | 181 ± 34.3^b^ | 220 ± 10.2^b^ | 308 ± 12.9^a^ | 250 ± 27.3^ab^ |
| Proline | 326 ± 19.5 | 212 ± 11.2^b^ | 190 ± 34.2^b^ | 223 ± 13^b^ | 306 ± 12.7^a^ | 246 ± 20.7^ab^ |
| Lysine | 477 ± 28.5 | 310 ± 18.9^b^ | 265 ± 61^b^ | 341 ± 14.2^ab^ | 455 ± 25^a^ | 374 ± 43^ab^ |
| Tyrosine | 223 ± 11.5 | 139 ± 8.68^b^ | 123 ± 27.3^b^ | 148 ± 8.09^b^ | 206 ± 8.26^a^ | 166 ± 17.8^ab^ |
| Methionine | 148 ± 7.08 | 98.3 ± 4.75^b^ | 98.9 ± 13.6^b^ | 101 ± 4.8^b^ | 142 ± 3.89^a^ | 110 ± 10.1^b^ |
| Valine | 348 ± 20.8 | 228 ± 11.2^b^ | 194 ± 39.4^b^ | 241 ± 10.6^b^ | 331 ± 13.3^a^ | 271 ± 27.7^ab^ |
| Isoleucine | 264 ± 16.2 | 170 ± 9.43^b^ | 157 ± 27.1^b^ | 181 ± 7.29^ab^ | 252 ± 11.1^a^ | 205 ± 23.1^ab^ |
| Leucine | 455 ± 27.5 | 295 ± 18.1^b^ | 259 ± 55.7^b^ | 320 ± 14.8^ab^ | 437 ± 19.7^a^ | 351 ± 37.1^ab^ |
| Phenylalanine | 292 ± 17.5 | 187 ± 11.8^b^ | 414 ± 157^ab^ | 203 ± 9.39^ab^ | 281 ± 11.5^a^ | 222 ± 25.6^ab^ |
| **TOTAL** | 6470 ± 363 | 4180 ± 255^b^ | 4040 ± 600^b^ | 4440 ± 194^b^ | 6090 ± 258^a^ | 4930 ± 537^ab^ |

# **Table C Fatty acid composition of initial samples of *Acropora millepora* and *Pocillopora acuta* (mg g lipid^-1^ and % fatty acids).** Values are presented as means ± SEM.

| **Fatty acids** | ***A. millepora*** | | ***P. acuta*** | |
| --- | --- | --- | --- | --- |
|  | *mg g lipid^-1^* | *% fatty acids* | *mg g lipid^-1^* | *% fatty acids* |
| 10:0 | 1.23 ± 0.71 | 1.06 ± 0.42 | 0.01 ± 0.02 | 0.02 ± 0.03 |
| 14:0 | 3.94 ± 2.44 | 3.19 ± 0.63 | 13.9 ± 8.4 | 5.47 ± 0.2 |
| 16:0 | 35 ± 20.5 | 29.3 ± 1.71 | 87.5 ± 54.3 | 34.2 ± 1.13 |
| 18:0 | 12.4 ± 7.09 | 11 ± 1.93 | 31.1 ± 18.9 | 12.5 ± 0.48 |
| **∑SFA** | 57.2 ± 32.9 | 48.7 ± 1.06 | 143 ± 87.4 | 56.8 ± 1.38 |
| 18:1n-9 | 6.21 ± 3.81 | 5.06 ± 0.55 | 12.4 ± 8.47 | 4.5 ± 0.79 |
| 16:OH | 9.79 ± 6.88 | 7.43 ± 2.67 | 17.7 ± 12.2 | 6.6 ± 0.75 |
| **∑MUFA** | 16.8 ± 10.5 | 14.1 ± 1 | 32.8 ± 21.4 | 12.2 ± 1.99 |
| 18:3n-3 | 0.04 ± 0.03 | 0.03 ± 0.02 | 0.23 ± 0.14 | 0.1 ± 0.02 |
| 18:4n-3 | 0 ± 0 | 0 ± 0 | 0 ± 0 | 0 ± 0 |
| 20:4n-3 | 0 ± 0 | 0 ± 0 | 1.46 ± 1.48 | 0.6 ± 0.23 |
| 20:5n-3 | 11.6 ± 6.13 | 10.5 ± 1.72 | 6.11 ± 3.25 | 2.83 ± 0.67 |
| 22:5n-3 | 3.75 ± 1.98 | 3.34 ± 0.32 | 1.46 ± 0.81 | 0.69 ± 0.14 |
| 22:6n-3 | 2.58 ± 1.72 | 2.21 ± 0.46 | 27.5 ± 17.8 | 10.5 ± 0.65 |
| 18:2n-6 | 1.24 ± 0.71 | 1.04 ± 0.13 | 1.82 ± 0.99 | 0.83 ± 0.17 |
| 18:3n-6 | 2.61 ± 1.83 | 2 ± 0.33 | 2.04 ± 1.31 | 0.77 ± 0.07 |
| 20:3n-6 | 0.87 ± 0.45 | 0.77 ± 0.06 | 6.55 ± 4.53 | 2.33 ± 0.39 |
| 20:4n-6 | 4.53 ± 2.57 | 3.99 ± 0.5 | 4.03 ± 2.24 | 1.86 ± 0.4 |
| 22:4n-6 | 4.88 ± 2.45 | 4.57 ± 0.88 | 4.74 ± 2.51 | 2.23 ± 0.49 |
| **∑PUFA** | 32.7 ± 18.1 | 29 ± 3.59 | 57.6 ± 35.2 | 23.4 ± 1.38 |
| **TOTAL** | 117 ± 67.7 | 100 ± 0 | 254 ± 157 | 100 ± 0 |
| **∑n-3 PUFA** | 18.1 ± 10 | 16.3 ± 2.45 | 37.2 ± 23.2 | 14.9 ± 1.08 |
| **∑n-6 PUFA** | 14.4 ± 8.05 | 12.7 ± 1.15 | 20.3 ± 12 | 8.4 ± 0.82 |
| **∑n-3 LC-PUFA** | 18.2 ± 10.1 | 16.3 ± 2.46 | 37.1 ± 23.2 | 14.9 ± 1.11 |
| **∑n-6 LC-PUFA** | 10.3 ± 5.44 | 9.34 ± 1.38 | 15.3 ± 8.94 | 6.42 ± 0.68 |

# **Table D Effect of five different feeding regimes on the fatty acid composition of *Acropora millepora* after 90 days (mg g lipid^-1^ and % fatty acids).** Values are presented as means ± SEM. Values in the same row that do not share a superscript are significantly different (*P*<0.05). n=3. Values in the same row that do not share a subscript are significantly different (*P*<0.05). ART = *Artemia* nauplii, ATF = artificial diet, CTL = ultra-filtered (0.04 μm) seawater, RAW = unfiltered seawater, RR = Reef Roids.

| **Fatty acids** | **ART** | | **ATF** | | **CTL** | | **RAW** | | **RR** | |
| --- | --- | --- | --- | --- | --- | --- | --- | --- | --- | --- |
|  | *mg g lipid^-1^* | *% fatty acids* | *mg g lipid^-1^* | *% fatty acids* | *mg g lipid^-1^* | *% fatty acids* | *mg g lipid^-1^* | *% fatty acids* | *mg g lipid^-1^* | *% fatty acids* |
| 10:0 | 1.48 ± 0.16^ab^ | 0.9 ± 0.2_a_ | 3.9 ± 2.16^ab^ | 0.5 ± 0.09_a_ | 3.33 ± 0.68^ab^ | 0.67 ± 0.15_a_ | 4.94 ± 0.08^b^ | 1.18 ± 0.13_a_ | 1.1 ± 0.35^a^ | 0.88 ± 0.3_a_ |
| 14:0 | 6.81 ± 0.4^a^ | 3.96 ± 0.18_cd_ | 10.2 ± 4.67^ab^ | 2.85 ± 0.22_a_ | 10.1 ± 1.66^ab^ | 3.19 ± 0.21_ab_ | 17.7 ± 1.22^b^ | 4.62 ± 0.07_d_ | 5.52 ± 0.56^a^ | 3.99 ± 0.46_bc_ |
| 16:0 | 61.3 ± 2.33^a^ | 31.8 ± 1.55_a_ | 73.6 ± 30.9^ab^ | 29.4 ± 1.52_a_ | 79.4 ± 13.1^ab^ | 30.7 ± 0.49_a_ | 141 ± 10.4^b^ | 36.6 ± 1.23_b_ | 51.5 ± 4.6^a^ | 30.7 ± 1.28_a_ |
| 18:0 | 17.4 ± 0.4^a^ | 8.63 ± 0.94_ab_ | 17.6 ± 5.86^a^ | 11.4 ± 0.63_b_ | 15.6 ± 3.01^a^ | 10.1 ± 0.5_b_ | 20.9 ± 2.46^a^ | 6.33 ± 0.77_a_ | 18.4 ± 2.15^a^ | 9.58 ± 1.19_ab_ |
| **∑SFA** | 94.9 ± 3.54^a^ | 50.2 ± 1.11_a_ | 118 ± 49.4^ab^ | 49.3 ± 1.91_a_ | 119 ± 19^ab^ | 49.7 ± 0.53_a_ | 196 ± 15^b^ | 52 ± 0.68_a_ | 83.2 ± 7.11^a^ | 50.1 ± 1.5_a_ |
| 18:1n-9 | 10.1 ± 0.81^a^ | 5.29 ± 0.2_ab_ | 15.7 ± 6.83^ab^ | 4.71 ± 0.2_a_ | 14.2 ± 2.67^ab^ | 5.27 ± 0.19_ab_ | 23.5 ± 0.76^b^ | 5.95 ± 0.16_b_ | 8.43 ± 0.67^a^ | 5.7 ± 0.41_b_ |
| 16:OH | 18.9 ± 2.19^a^ | 11.3 ± 0.71_bc_ | 36.5 ± 18.4^ab^ | 6.94 ± 0.66_a_ | 35 ± 6.65^ab^ | 8.4 ± 1.17_ab_ | 61.5 ± 3.72^b^ | 15.2 ± 1_c_ | 13.5 ± 2.73^a^ | 12.6 ± 2.38_bc_ |
| **∑MUFA** | 16 ± 1.04^a^ | 8.63 ± 0.44_ab_ | 23.1 ± 9.66^ab^ | 8.08 ± 0.07_a_ | 21.2 ± 3.61^ab^ | 8.53 ± 0.21_ab_ | 33.7 ± 1.45^b^ | 8.73 ± 0.03_ab_ | 14 ± 0.9^ab^ | 8.94 ± 0.54_b_ |
| 18:3n-3 | 0.11 ± 0^ab^ | 0.12 ± 0.07_a_ | 0.09 ± 0.03^ab^ | 0.05 ± 0.01_a_ | 0.1 ± 0.02^ab^ | 0.06 ± 0.01_a_ | 0.15 ± 0.01^b^ | 0.04 ± 0.01_a_ | 0.07 ± 0.01^a^ | 0.06 ± 0.01_a_ |
| 18:4n-3 | 5.04 ± 0.2^a^ | 2.38 ± 0.16_ab_ | 5.17 ± 2.03^a^ | 3.19 ± 0.1_c_ | 5.11 ± 0.59^a^ | 2.82 ± 0.1_bc_ | 8.21 ± 0.57^a^ | 2.3 ± 0.12_a_ | 4.9 ± 0.33^a^ | 2.17 ± 0.2_a_ |
| 20:4n-3 | 0.22 ± 0.04^a^ | 0.16 ± 0.03_a_ | 0.32 ± 0.14^ab^ | 0.14 ± 0.05_a_ | 0.35 ± 0.08^ab^ | 0.09 ± 0.01_a_ | 0.59 ± 0.04^b^ | 0.23 ± 0.07_a_ | 0.24 ± 0.02^a^ | 0.24 ± 0.14_a_ |
| 20:5n-3 | 15.3 ± 0.57^a^ | 6.62 ± 0.99_a_ | 15.1 ± 5.61^a^ | 10.2 ± 0.83_c_ | 14.4 ± 1.07^a^ | 8.92 ± 0.32_bc_ | 17.6 ± 1.23^a^ | 4.76 ± 0.15_a_ | 16.5 ± 1.22^a^ | 6.1 ± 1.05_ab_ |
| 22:5n-3 | 5.72 ± 0.23^a^ | 2.55 ± 0.41_ab_ | 6.86 ± 2.88^a^ | 3.42 ± 0.18_c_ | 6.34 ± 0.72^a^ | 3.19 ± 0.05_bc_ | 7.73 ± 0.42^a^ | 1.99 ± 0.11_a_ | 5.63 ± 0.36^a^ | 2.45 ± 0.36_ac_ |
| 22:6n-3 | 3.92 ± 0.06^a^ | 2.95 ± 0.68_a_ | 2.83 ± 0.83^a^ | 2.41 ± 0.12_a_ | 3.67 ± 0.63^a^ | 2.13 ± 0.14_a_ | 6.84 ± 0.86^b^ | 2.73 ± 0.85_a_ | 3.75 ± 0.37^ab^ | 3.35 ± 1.5_a_ |
| 18:2n-6 | 2.1 ± 0.14^a^ | 1.04 ± 0.09_ab_ | 2.49 ± 1.03^a^ | 1.04 ± 0.04_ab_ | 2.44 ± 0.41^a^ | 1.15 ± 0.02_b_ | 3.37 ± 0.15^a^ | 0.86 ± 0.03_a_ | 1.96 ± 0.3^a^ | 0.96 ± 0.05_ab_ |
| 18:3n-6 | 4.65 ± 0.25^a^ | 2.28 ± 0.18_ab_ | 6.3 ± 3^a^ | 1.95 ± 0.14_a_ | 6.78 ± 1.12^ab^ | 2.29 ± 0.1_ab_ | 13.4 ± 0.71^b^ | 3.23 ± 0.35_b_ | 3.52 ± 0.33^a^ | 2.07 ± 0.34_a_ |
| 20:3n-6 | 1.31 ± 0.06^a^ | 1.05 ± 0.22_a_ | 1.73 ± 0.57^a^ | 0.87 ± 0.1_a_ | 1.52 ± 0.24^a^ | 0.8 ± 0.07_a_ | 2.12 ± 0.21^a^ | 0.76 ± 0.19_a_ | 1.36 ± 0.15^a^ | 1.15 ± 0.3_a_ |
| 20:4n-6 | 5.81 ± 0.32^a^ | 2.84 ± 0.4_b_ | 5.89 ± 1.92^a^ | 4.17 ± 0.4_c_ | 5.53 ± 0.71^a^ | 3.64 ± 0.12_bc_ | 5.4 ± 0.35^a^ | 1.55 ± 0.05_a_ | 6.67 ± 1.01^a^ | 2.69 ± 0.55_b_ |
| 22:4n-6 | 5.84 ± 0.17^a^ | 2.76 ± 0.46_b_ | 5.89 ± 1.98^a^ | 4.24 ± 0.37_c_ | 5.09 ± 0.48^a^ | 3.69 ± 0.18_bc_ | 4.61 ± 0.27^a^ | 1.42 ± 0.12_a_ | 6.77 ± 0.92^a^ | 2.77 ± 0.6_b_ |
| **∑PUFA** | 53.1 ± 2.16^a^ | 26.9 ± 1.84_b_ | 55.7 ± 20.7^a^ | 33.7 ± 1.98_c_ | 55.2 ± 4.33^a^ | 30.9 ± 1.01_bc_ | 73 ± 4.92^a^ | 20.8 ± 0.93_a_ | 54.6 ± 4.43^a^ | 25.5 ± 2.33_ab_ |
| **TOTAL** | 188 ± 8.93^a^ | 100 ± 0_a_ | 239 ± 99.2^ab^ | 100 ± 0_b_ | 238 ± 35.2^ab^ | 100 ± 0_b_ | 377 ± 24.9^b^ | 100 ± 0_b_ | 170 ± 11.9^ab^ | 100 ± 0_b_ |
| **∑n-3 PUFA** | 30.8 ± 1.11^a^ | 15 ± 1.22_ab_ | 31.1 ± 11.6^a^ | 19.8 ± 1.17_c_ | 30.4 ± 2.51^a^ | 17.6 ± 0.58_bc_ | 41.4 ± 3.14^a^ | 12.2 ± 0.88_a_ | 31.6 ± 2.21^a^ | 14.7 ± 1.31_ab_ |
| **∑n-6 PUFA** | 20.6 ± 0.93^a^ | 10.4 ± 0.66_b_ | 23.2 ± 8.63^a^ | 13 ± 0.74_c_ | 22.2 ± 2.72^a^ | 12.3 ± 0.29_bc_ | 30 ± 1.7^a^ | 8.16 ± 0.01_a_ | 21.4 ± 2.4^a^ | 10.1 ± 1.1_b_ |
| **∑n-3 LC-PUFA** | 25.7 ± 0.91^a^ | 12.5 ± 1.04_ab_ | 25.8 ± 9.57^a^ | 16.6 ± 1.09_c_ | 25.2 ± 1.92^a^ | 14.7 ± 0.5_bc_ | 33.1 ± 2.56^a^ | 9.84 ± 0.76_a_ | 26.7 ± 1.9^a^ | 12.5 ± 1.18_ab_ |
| **∑n-6 LC-PUFA** | 13 ± 0.54^a^ | 6.67 ± 0.66_bc_ | 13.5 ± 4.48^a^ | 9.32 ± 0.83_b_ | 12.2 ± 1.33^a^ | 8.15 ± 0.37_bc_ | 12.2 ± 0.82^a^ | 3.73 ± 0.36_a_ | 14.9 ± 2.07^a^ | 6.61 ± 1.16_c_ |

# **Table E Effect of five different feeding regimes on the fatty acid composition of *Pocillopora acuta* after 90 days (mg g lipid^-1^ and % fatty acids).** Values are presented as means ± SEM. Values in the same row that do not share a superscript are significantly different (*P*<0.05). n=3. Values in the same row that do not share a subscript are significantly different (*P*<0.05). ART = *Artemia* nauplii, ATF = artificial diet, CTL = ultra-filtered (0.04 μm) seawater, RAW = unfiltered seawater, RR = Reef Roids.

| **Fatty acids** | **ART** | | **ATF** | | **CTL** | | **RAW** | | **RR** | |
| --- | --- | --- | --- | --- | --- | --- | --- | --- | --- | --- |
|  | *mg g lipid^-1^* | *% fatty acids* | *mg g lipid^-1^* | *% fatty acids* | *mg g lipid^-1^* | *% fatty acids* | *mg g lipid^-1^* | *% fatty acids* | *mg g lipid^-1^* | *% fatty acids* |
| 10:0 | 0.04 ± 0.03^a^ | 0.26 ± 0.26_a_ | 0.04 ± 0.01^a^ | 0.02 ± 0.01_a_ | 0.01 ± 0.01^a^ | 0.17 ± 0.15_a_ | 0.09 ± 0.04^a^ | 0.07 ± 0.03_a_ | 0.04 ± 0.02^a^ | 0.22 ± 0.22_a_ |
| 14:0 | 16.4 ± 2.09^a^ | 4.69 ± 0.23_a_ | 22.5 ± 4.75^a^ | 4.59 ± 0.07_a_ | 16.4 ± 4.82^a^ | 4.74 ± 0.14_a_ | 17 ± 2.83^a^ | 4.22 ± 0.82_a_ | 10.8 ± 2.98^a^ | 4.9 ± 0.18_a_ |
| 16:0 | 105 ± 14.8^a^ | 33.7 ± 1.01_ab_ | 151 ± 30.7^a^ | 33.3 ± 0.48_ab_ | 119 ± 35.2^a^ | 31 ± 0.38_a_ | 113 ± 19.7^a^ | 33.4 ± 1.16_ab_ | 77.4 ± 21.3^a^ | 36.3 ± 2.3_b_ |
| 18:0 | 42.4 ± 5.92^a^ | 11.2 ± 0.92_a_ | 53.9 ± 10.8^a^ | 12.9 ± 0.29_a_ | 35.3 ± 7.76^a^ | 12.2 ± 0.47_a_ | 39.7 ± 7^a^ | 12.3 ± 0.12_a_ | 28.5 ± 7.79^a^ | 10.4 ± 1.81_a_ |
| **∑SFA** | 185 ± 27.6^a^ | 54.8 ± 2.61_a_ | 248 ± 50.6^a^ | 56.8 ± 0.75_a_ | 188 ± 52.5^a^ | 54.8 ± 0.68_a_ | 188 ± 32.4^a^ | 55.2 ± 2.05_a_ | 128 ± 34.8^a^ | 56.1 ± 0.67_a_ |
| 18:1n-9 | 12.9 ± 2.35^a^ | 4.42 ± 0.38_a_ | 20.8 ± 4.3^a^ | 4.22 ± 0.12_a_ | 18.9 ± 6.59^a^ | 4.12 ± 0.32_a_ | 13.4 ± 2.53^a^ | 3.96 ± 0.14_a_ | 9.91 ± 2.66^a^ | 4.74 ± 0.3_a_ |
| 16:OH | 24.5 ± 4.33^a^ | 10.6 ± 0.81_a_ | 41 ± 9.63^a^ | 7.85 ± 0.96_a_ | 32.6 ± 9.77^a^ | 7.81 ± 0.45_a_ | 28.2 ± 4.73^a^ | 7.65 ± 1.41_a_ | 20.3 ± 6.22^a^ | 10.2 ± 1.13_a_ |
| **∑MUFA** | 24.9 ± 5.04^a^ | 7.77 ± 0.6_a_ | 39 ± 7.6^a^ | 7.52 ± 0.28_a_ | 35.9 ± 13.2^a^ | 7.76 ± 0.56_a_ | 24.9 ± 4.89^a^ | 7.27 ± 0.31_a_ | 18 ± 4.84^a^ | 8.25 ± 0.21_a_ |
| 18:3n-3 | 0.72 ± 0.12^b^ | 0.11 ± 0.03_a_ | 0.5 ± 0.09^ab^ | 0.13 ± 0.02_a_ | 0.44 ± 0.13^ab^ | 0.17 ± 0.03_a_ | 0.32 ± 0.06^a^ | 0.09 ± 0.02_a_ | 0.24 ± 0.06^a^ | 0.09 ± 0.03_a_ |
| 18:4n-3 | 9.39 ± 1.29^a^ | 1.91 ± 0.24_a_ | 7.94 ± 1.22^a^ | 2.25 ± 0.13_ab_ | 4.89 ± 1.31^a^ | 3.06 ± 0.12_b_ | 7.87 ± 1.21^a^ | 2.54 ± 0.23_ab_ | 4.41 ± 1.1^a^ | 2.08 ± 0.23_a_ |
| 20:4n-3 | 1.66 ± 0.11^a^ | 0.64 ± 0.13_a_ | 2.63 ± 0.82^a^ | 0.45 ± 0.11_a_ | 1.62 ± 0.14^a^ | 0.44 ± 0.13_a_ | 2.79 ± 0.47^a^ | 0.62 ± 0.25_a_ | 1.32 ± 0.48^a^ | 0.45 ± 0.16_a_ |
| 20:5n-3 | 12.3 ± 2.37^b^ | 3.59 ± 1.72_a_ | 8.34 ± 1.27^ab^ | 2.74 ± 0.23_a_ | 4.7 ± 1.24^ab^ | 4.47 ± 0.64_a_ | 8.51 ± 1.15^ab^ | 5.08 ± 2.52_a_ | 5.07 ± 1.29^a^ | 3.15 ± 1.06_a_ |
| 22:5n-3 | 4.03 ± 1.05^a^ | 1.67 ± 0.35_a_ | 2.94 ± 0.57^a^ | 2.85 ± 0.46_a_ | 1.85 ± 0.53^a^ | 2.16 ± 0.11_a_ | 2.48 ± 0.38^a^ | 2.35 ± 0.86_a_ | 1.64 ± 0.51^a^ | 1.37 ± 0.13_a_ |
| 22:6n-3 | 30.6 ± 4.11^ab^ | 1.69 ± 0.4_ab_ | 43.4 ± 8.64^b^ | 2.73 ± 0.2_ab_ | 29.6 ± 6.42^ab^ | 2.95 ± 0.14_b_ | 30.8 ± 4.87^ab^ | 2.52 ± 0.84_ab_ | 18.5 ± 5.01^a^ | 1.39 ± 0.09_a_ |
| 18:2n-6 | 2.88 ± 0.58^a^ | 1.36 ± 0.66_a_ | 2.79 ± 0.56^a^ | 0.8 ± 0.06_a_ | 2.27 ± 0.61^a^ | 1.52 ± 0.27_a_ | 2.06 ± 0.42^a^ | 1.66 ± 0.88_a_ | 1.72 ± 0.45^a^ | 1.12 ± 0.4_a_ |
| 18:3n-6 | 2.53 ± 0.4^a^ | 8.25 ± 1.93_a_ | 4.07 ± 0.76^a^ | 7.45 ± 0.46_a_ | 3.59 ± 1.06^a^ | 8.19 ± 1.11_a_ | 2.88 ± 0.56^a^ | 7.26 ± 2.34_a_ | 1.91 ± 0.47^a^ | 7.51 ± 2.4_a_ |
| 20:3n-6 | 6.43 ± 1.1^a^ | 0.82 ± 0.07_a_ | 12.9 ± 2.47^a^ | 1.07 ± 0.22_a_ | 11.5 ± 3.76^a^ | 0.86 ± 0.05_a_ | 7.61 ± 1.37^a^ | 0.83 ± 0.13_a_ | 5.22 ± 1.32^a^ | 0.72 ± 0.06_a_ |
| 20:4n-6 | 6.54 ± 1.33^a^ | 1.32 ± 0.3_a_ | 4.96 ± 0.67^a^ | 0.94 ± 0.05_a_ | 2.86 ± 0.66^a^ | 0.96 ± 0.16_a_ | 4.41 ± 0.56^a^ | 1.18 ± 0.31_a_ | 4.29 ± 1.06^a^ | 1.75 ± 0.83_a_ |
| 22:4n-6 | 9.36 ± 2.16^b^ | 2.17 ± 0.54_a_ | 5.5 ± 0.91^ab^ | 2.02 ± 0.15_a_ | 2.84 ± 0.66^a^ | 1.77 ± 0.23_a_ | 5.46 ± 0.93^ab^ | 1.88 ± 0.42_a_ | 4.54 ± 1.27^ab^ | 2.09 ± 0.74_a_ |
| **∑PUFA** | 92.1 ± 13.4^a^ | 24.6 ± 1.11_a_ | 101 ± 18.9^a^ | 25.4 ± 0.79_a_ | 74.1 ± 21.4^a^ | 28.4 ± 0.3_a_ | 79.6 ± 12.8^a^ | 27.7 ± 3.24_a_ | 52.6 ± 13.7^a^ | 22.8 ± 1.2_a_ |
| **TOTAL** | 332 ± 45.3^a^ | 100 ± 0_a_ | 441 ± 88.8^a^ | 100 ± 0_b_ | 338 ± 98.5^a^ | 100 ± 0_b_ | 327 ± 55.4^a^ | 100 ± 0_b_ | 225 ± 60.8^a^ | 100 ± 0_b_ |
| **∑n-3 PUFA** | 59.7 ± 8.34^a^ | 16.1 ± 0.62_ab_ | 66.8 ± 12.6^a^ | 14.2 ± 0.2_a_ | 43.7 ± 9.6^a^ | 18.1 ± 0.3_b_ | 53.7 ± 8.23^a^ | 17.6 ± 1.4_ab_ | 32 ± 8.61^a^ | 14.6 ± 1.11_a_ |
| **∑n-6 PUFA** | 29.4 ± 4.66^a^ | 8.1 ± 0.52_a_ | 32.3 ± 5.87^a^ | 10.2 ± 0.84_a_ | 24.7 ± 7.3^a^ | 9.21 ± 0.2_a_ | 23.7 ± 4.05^a^ | 9.25 ± 1.78_a_ | 19.1 ± 4.79^a^ | 7.71 ± 0.29_a_ |
| **∑n-3 LC-PUFA** | 49.6 ± 7.01^ab^ | 14 ± 0.41_a_ | 58.3 ± 11.3^b^ | 11.9 ± 0.33_a_ | 38.4 ± 8.19^ab^ | 14.9 ± 0.34_a_ | 45.5 ± 6.97^ab^ | 15 ± 1.21_a_ | 27.4 ± 7.49^a^ | 12.4 ± 1.21_a_ |
| **∑n-6 LC-PUFA** | 22.5 ± 3.63^a^ | 5.54 ± 0.22_ab_ | 23.4 ± 4.04^a^ | 7.64 ± 0.53_b_ | 17.2 ± 5.07^a^ | 6.89 ± 0.08_ab_ | 17.6 ± 2.87^a^ | 6.77 ± 1.28_ab_ | 14.4 ± 3.7^a^ | 4.86 ± 0.72_a_ |
